# Low-Cost CO_2_ Sensors: On-Site Performance Evaluation and Co-Location Correction Procedure for Reliable Ventilation Assessments in Schools

**DOI:** 10.3390/s26041265

**Published:** 2026-02-15

**Authors:** David Honan, John Garvey, John Littlewood, Matthew Horrigan, John Gallagher

**Affiliations:** 1 Department of Civil, Structural & Environmental Engineering, Trinity College Dublin, The University of Dublin, D02 PN40 Dublin, Ireland; j.gallagher@tcd.ie; 2 Department of the Built Environment, Technological University of the Shannon, V94 EC5T Limerick, Ireland; 3 Kemmy Business School, University of Limerick, V94 T9PX Limerick, Ireland; john.garvey@ul.ie; 4 The Sustainable & Resilient Built Environment Research Group, Cardiff School of Art & Design, Cardiff Metropolitan University, Cardiff CF5 2YB, UK; jlittlewood@cardiffmet.ac.uk; 5 Department of Business and Financial Services, Technological University of the Shannon, V94 EC5T Limerick, Ireland; matthew.horrigan@tus.ie; 6 TrinityHaus Trinity Research Centre, Trinity College Dublin, The University of Dublin, D02 PN40 Dublin, Ireland

**Keywords:** low-cost CO_2_ sensors, CO_2_ measurement, performance evaluation, co-location normalisation, ventilation assessment, school classrooms, Ireland

## Abstract

Adequate ventilation is essential for maintaining indoor environmental quality in schools, where ventilation standards are often based on an indoor concentration of human-generated carbon dioxide (CO_2_) above ambient levels. Low-cost non-dispersive infrared (NDIR) CO_2_ sensors offer a practical solution for ventilation monitoring, yet variability between sensors can compromise accuracy, particularly when applications depend on the determination of precise concentration differences. This study evaluates the performance of twenty-three low-cost CO_2_ sensors, developing normalisation functions to improve comparability across sensors, introducing an accessible methodology for on-site sensor calibration without the need for laboratory-grade reference equipment. The sensors were co-located for three independent test periods in 2025 representing typical school internal conditions in Ireland. Pre-normalisation analysis showed strong linearity (coefficient of determination (R^2^) = 0.999) but notable variability, with a mean root mean square error (RMSE) of 18.3 ppm and 0.45% of measurements outside manufacturers stated accuracy. Normalisation models were trained and validated using a leave-one-period-out approach. Regression-based correction yielded the greatest improvement, reducing RMSE by 16%. When applied to the full dataset, final correction factors reduced RMSE by 27%, out-of-range measurements by 43%, and proportional bias by 31%. Corrected sensors demonstrated highly consistent performance, particularly within the CO_2_ ranges most relevant for classroom ventilation assessment, with an RMSE = 7.4 parts per million (ppm) at ambient concentrations and 11.9 ppm at concentrations below 1500 ppm. Field-based co-location in the deployment environment across full CO_2_ cycles, combined with a network-derived global reference, produced effective correction factors. Performance declined marginally above 1500 ppm and during dynamic occupancy, while overall accuracy remained strong. The study presents a practical and accessible methodology for evaluating and normalising low-cost CO_2_ sensors without specialised laboratory equipment, supporting reliable ventilation assessments in schools.

## 1. Introduction

This study evaluates the reliability of low-cost (carbon dioxide) CO_2_ sensors and introduces an innovative, practical methodology for on-site calibration that does not require laboratory-grade equipment or complex machine learning techniques, thereby supporting reliable CO_2_-based ventilation assessments in schools. Ventilation is a critical component of classroom indoor environmental quality (IEQ), directly influencing occupant health and learning performance [1,2,3]. Human-generated CO_2_ is commonly employed as a tracer gas for estimating ventilation rates (VRs) and as a surrogate indicator of ventilation adequacy [4,5]. Both the American Society of Heating, Refrigerating and Air-Conditioning Engineers (ASHRAE) and European Standards define limit values for CO_2_ as “maximum above ambient” based on the difference between indoor and outdoor (I/O) CO_2_ concentrations (ΔCO_2_) [5]. For example, the EN 16798-1 category 1 limit for classrooms is set at ≤550 parts per million (ppm) above ambient levels [6].

In naturally ventilated (NV) classrooms, where VRs are influenced by occupant behaviour, window operation, and external environmental conditions, continuous CO_2_ monitoring provides an effective means of assessing ventilation adequacy and managing indoor air quality (IAQ) [7,8]. Additionally, continuous CO_2_ monitoring can support the implementation and evaluation of ventilation strategies aimed at reducing occupancy-related pollutant loading in classrooms, thereby improving comfort and reducing airborne transmission risks [9,10].

CO_2_ monitoring for ventilation adequacy has become more prevalent in schools since the COVID-19 pandemic [11]. Low-cost non-dispersive infrared (NDIR) CO_2_ sensors are a popular choice for schools due to their affordability, user-friendly design and portability. NDIR sensors operate by measuring the absorption of infrared light at specific wavelengths characteristic of CO_2_, with the amount of absorbed light directly related to the gas concentration via the Beer–Lambert law [12].

Networked CO_2_ sensors offer further benefits, enabling centralised data logging and remote monitoring, allowing school management to compare conditions across multiple spaces and identify periods of under-ventilation in real time [13]. In addition, networked sensor systems provide a scalable and low-cost mechanism for collecting the longitudinal evidence needed to demonstrate compliance with national and international ventilation and IAQ requirements in schools [11], for which inter-device consistency and the reliability of data collection are paramount.

The accuracy of low-cost NDIR CO_2_ sensors is strongly influenced by calibration procedures [14]. Furthermore, inter-sensor variability, the degree to which sensors differ from one another, can vary considerably. For instance, Dubey et al. [15] reported root mean square error (RMSE) values ranging from 131 ppm to 6 ppm and R^2^ values from 0.71 to 0.98 when comparing nine low-cost sensors against a reference instrument.

The reliability of ventilation adequacy assessments, typically based on the I/O ΔCO_2_, is further affected by the CO_2_ sampling approach. Although single-point indoor measurements and reference outdoor values are frequently used in both practice and research [3,16], CO_2_ concentrations within classrooms can exhibit substantial spatial variability [11]. Studies have observed spatial variations of up to 242 ppm within occupied classrooms [17], highlighting the need for multi-point CO_2_ sampling to more accurately represent room-average conditions [8,16]. In addition, outdoor CO_2_ levels are not constant, varying by location and season due to meteorological and biospheric processes, with transient concentrations in some cases exceeding 600 ppm, which is well above the 2024 global mean of 426 ppm [5]. Consequently, accurate local outdoor measurements are essential when using CO_2_ as a tracer gas for estimating VRs.

Given these factors, the accurate determination of ΔCO_2_ and mean indoor CO_2_ concentrations, where multiple sensors are employed, is dependent on inter-sensor reliability, that is, whether sensors respond consistently to the same environmental conditions. Beyond standard calibration processes, the application of co-location normalisation and bias-correction procedures along with the evaluation and reporting of inter-device performance are critical to ensure data reliability and support accurate VR estimation in practice [18].

The accuracy and reliability of NDIR sensors is influenced by several factors, including optical path length, environmental interferences (temperature, humidity, pressure), cross-sensitivity to other gases, and sensor drift [19,20,21]. Recent advances have focused on improving compensation mechanisms, such as temperature and humidity correction algorithms, and optimising sensor design to minimise errors and extend applicability in real-world settings [19,22,23].

Once frequently calibrated and appropriately corrected, NDIR sensors can produce highly comparable data, at least in a relative sense, making them suitable for use in ventilation and air quality studies [22,24]. Despite this, many studies employing low-cost NDIR sensors for classroom CO_2_ monitoring do not assess or report inter-device performance, nor do they adequately describe or implement calibration and correction procedures [16,25]. Without appropriate calibration and normalisation, raw CO_2_ measurements cannot be directly compared across devices, thereby undermining the accuracy of VR estimation, spatial mapping, and the reliability of subsequent analyses. Sensor co-location and statistical normalisation are therefore essential methodological steps to harmonise sensor responses, enabling robust inter-sensor comparison and the generation of reliable, comparable datasets.

Co-location of low-cost NDIR CO_2_ sensors with a common reference instrument is a well-established and effective method for ensuring the validity and comparability of measurements across sensor networks [26]. This approach enables the correction of sensor drift and adjustment for environmental influences under real-world conditions, thereby improving CO_2_ measurement accuracy and reducing systematic bias [22]. Calibration and correction based on linear regression have been shown to improve RMSE by up to 62% for low-cost NDIR CO_2_ sensors [15]. Muller et al. [14] reported accuracies between 8 ppm and 12 ppm over deployment periods of 19 to 25 months for carefully calibrated low-cost NDIR sensors operating under ambient conditions. Although machine learning approaches are increasingly used for sensor correction, Dubey et al. [15] found that simple linear regression outperformed more complex models such as gradient boosting and random forest regression.

Recent standards and working-group initiatives recognise persistent uncertainties in the reliability of low-cost CO_2_ measurements and propose chamber-based test methods for evaluating sensor performance against reference instruments [5,27]. Yet empirical evidence from real classroom environments remains limited, particularly regarding effective approaches for normalising sensor outputs for relative comparison. Most calibration studies report co-location results obtained with respect to reference instrumentation under controlled laboratory conditions [26,28]. However, for classroom ventilation assessments the inter-sensor ΔCO_2_ is more relevant than absolute concentrations. Furthermore, access to reference-grade instruments, environmental test chambers, and advanced data-processing or machine learning capabilities is typically beyond the reach of schools and other non-specialist users.

This study adopts a network-derived global reference (GR), defined as the median response of co-located sensors, which aligns conceptually with consensus-based approaches reported in the literature (e.g., Smith et al. [29]). This ensemble-based method is well suited to evaluating inter-sensor consistency, identifying drift and medium-term response divergence, and reducing the influence of anomalous devices where a single trusted reference is unavailable, thereby providing a robust framework for comparative sensor performance assessment in field settings [29].

The aim of this study is to provide field-based empirical evidence on the accuracy and reliability of low-cost CO_2_ sensors while demonstrating a practical, novel methodology for on-site calibration and normalisation. Specifically, the study seeks to leverage a global reference (GR) combined with accessible tools, such as simple regression techniques, to correct and normalise sensor measurements without relying on laboratory-grade instruments or complex machine learning methods. This approach establishes a scalable framework for co-location calibration and real-world performance evaluation of low-cost NDIR sensors, enabling reliable CO_2_-based ventilation assessments in schools. As a result, the study enhances confidence in the practical application of co-location procedures in school settings supporting classroom monitoring, spatial mapping, and ventilation analysis, and mitigating the risks associated with uncorrected sensor drift.

## 2. Methods

The methodological approach developed for this study includes several steps: (i) evaluating pre-normalisation sensor performance relative to a global reference (GR); (ii) training, testing, and validating normalisation models; (iii) deriving correction factors and assessing the performance of post-normalisation measurements; (iv) examining the influence of co-location test conditions and setups to identify potential confounding factors, ultimately providing recommendations and practical guidance for co-location testing in educational settings.

### 2.1. Co-Location Setup and Sampling

Twenty-three Aranet4 (SAF Tehnika, Riga, Latvia) low-cost wireless sensors [30] were deployed side-by-side for each co-location period (CP). All sensors were calibrated as per the manufacturer’s instructions [31] and time-synchronised prior to installation and configured to sample at 1-min intervals. All sensors were powered on and allowed a warm-up period of at least 30 min before data collection to ensure stable readings. Data from each sensor was transmitted in real time to an Aranet Pro base station [32], which served as the central data logger and storage hub for all devices. This configuration enabled continuous monitoring and ensured consistent timestamp alignment across the dataset. Minor differences in sensor response times within each 1-min timestamp cannot be discounted and may contribute to discrepancies, particularly during periods of rapidly changing CO_2_ concentrations, but these effects are expected to be minimal.

Co-location sampling was carried out during three periods over six months in 2025 to capture indoor environmental conditions across the heating (CP1), shoulder (CP2), and non-heating (CP3) seasons. Table 1 reports the mean, median, standard deviation (SD), and range of indoor environmental parameters for each CP. All sampling took place in NV spaces operating under normal occupancy schedules, enabling evaluation of sensor performance under realistic ranges of temperature, RH, and CO_2_ for NV school settings. To objectively assess how typical classroom co-location conditions affect sensor performance, different heights and locations, reflecting those most reported in the literature, were used across CP1, CP2, and CP3.

CP1 took place in a 27 m^3^ office over twenty-four hours in January 2025. Sensors were co-located 1.5 m above floor level, consistent with the sampling height recommended in EN ISO 16000-26 for indoor CO_2_ measurements [33]. The space was partially occupied by two adults, with windows and doors kept closed throughout to maintain stable indoor conditions.

CP2 was conducted over a seven-hour school day in March 2025 in a 288 m^3^ classroom occupied by twenty-eight pupils (average age: 11 years) and one teacher. The sampling period included occupied hours only, capturing the short-term variability in CO_2_ concentrations associated with occupancy patterns and NV events (i.e., manual opening of windows and doors at the teacher’s discretion). Sensors were co-located at the front of the classroom at a height of 0.8 m, representing the seated breathing zone of the pupils [34].

CP3 took place in the same classroom over forty-four hours in June 2025, including occupied and unoccupied hours to capture diurnal and occupancy-related variability. Sensors were co-located near an internal wall at a height of 2.2 m, corresponding to upper-level CO_2_ sampling heights used in previous classroom studies (e.g., Muelas et al. [35]).

### 2.2. Determination of Global Reference (GR)

Rather than referencing a single high-accuracy instrument, the median of the twenty-three synchronised sensors was adopted as a surrogate GR value for each timestamp. This approach was chosen for methodological and practical reasons, considering the application of the sensor network.

First, the primary objective of this study is to evaluate the relative performance of low-cost CO_2_ sensors and to normalise sensor measurements for the assessment of the spatial distribution of CO_2_ in a classroom environment, rather than to establish absolute CO_2_ concentrations. For spatial analyses, the relative deviation of each sensor from the network central tendency provides more meaningful insight into spatial consistency than comparison to an external standard.

Second, the median is a robust measure of central tendency that reduces the influence of localised disturbances, such as occupant breath plumes or sensor noise, which can disproportionately affect mean values or single-instrument references. Using the median therefore ensures that transient or outlier measurements do not bias the reference concentration. The median-based global reference assumes that sensor biases are approximately symmetric across the network. If multiple sensors shared a systematic bias, the median GR would also be biassed, potentially affecting residuals and correction accuracy. In this study, no evidence of strong shared bias was observed, supporting the median GR as a practical reference for co-located sensors.

Third, subsequent analyses such as estimation of VRs and spatial deviation metrics are based primarily on the difference in I/O and inter-sensor ΔCO_2_ respectively, rather than absolute levels. Furthermore, since the ASHRAE and EN standard IDA thresholds are defined as above ambient outdoor levels, the relative I/O inter-sensor difference is more critical than the absolute accuracy of the measurement. The network median thus provides a stable and representative baseline for evaluating sensor deviations relevant to the study’s objectives.

Finally, practical considerations and the future application of this methodology, considering school resources, support this approach. Firstly, continuous co-location of all sensors with a laboratory-grade reference over the full six-month sampling campaign was not feasible. Additionally, schools may not have access to laboratory-grade reference instruments. Therefore, the development of network-based references allows for consistent comparison across the sensor network in the context of classroom CO_2_ assessment.

### 2.3. Data Analysis

#### 2.3.1. Quality Control and Data Pre-Processing

All data was processed with Microsoft 365 Excel (Microsoft Corporation, 2022). The raw sensor outputs were first aligned by timestamp, resulting in 4488 one-minute records per sensor (103,224 data points in total). Any timestamp with missing data from one or more sensors was excluded (1034 missing values across 929 rows), leaving 81,857 valid data points (79%). This ensures that all analyses are based exclusively on complete sets of co-located measurements.

Extreme outliers were defined as values ±300 ppm from the row median in the time series sensor data. Extreme outliers (*n* = 5) were identified, all within a 3-min timeframe in CP1 (potentially due to the proximity of occupant breath plumes hitting the nearest sensors first) and replaced with the mean of the values immediately above and below (temporal interpolation), which preserved continuity without distorting short-term trends.

#### 2.3.2. Statistical Performance Metrics

A suite of statistical performance metrics was employed to provide a robust and multidimensional assessment of sensor performance. Table S1 of the Supplementary Materials summarises these performance metrics, outlining their mathematical formulations, analytical interpretation, and indicative thresholds for evaluating sensor performance.

While many existing evaluations of low-cost sensors focus primarily on the coefficient of determination (R^2^), this metric alone provides limited insight into absolute accuracy and bias. For example, Kang et al. [28] found that R^2^ was reported in 82% of studies, whereas RMSE and mean absolute error (MAE) were reported far less frequently (34% and 27%, respectively), while standard error of the estimate (SEE), which provides a useful indication of the consistency of model fit, was rarely included (2%). Similarly, Rai et al. [36] demonstrated that meaningful performance evaluation requires consideration of both goodness-of-fit and error magnitude, recommending the combined use of RMSE, MAE, and the coefficient of variation (CV) across diverse environmental conditions and sensor types. Mean Bias Error (MBE) was used in this study to quantify the direction and magnitude of systematic error between the low-cost sensors and the reference instrument. Unlike MAE, which removes sign information by taking the absolute value of differences, MBE preserves whether the sensor tends to overestimate or underestimate CO_2_ concentrations relative to the reference. MBE therefore provides a more informative metric for calibration and normalisation, particularly where sensor drift or offset effects are present [37]. MBE was prioritised in this study to support the identification of systematic bias and to guide the development of regression-based correction factors. Overall, the inclusion of RMSE, MBE, R^2^, SEE, and the CV in this study aligns with recommended practice and supports a balanced evaluation of precision, accuracy, and consistency.

To assess the sensor performance relative to manufacturer expectations, the percentage of deviations from stated accuracy specifications were quantified by analysing both the magnitude and frequency of departures from the GR across all timestamps. This approach enabled the identification of systematic offset, sensor drift, and condition-specific performance limitations relevant to each of the real-world co-location monitoring setups.

#### 2.3.3. Normalisation Model Development and Validation

While advanced machine learning techniques are increasingly employed for sensor correction [28], this study adopted simple bias offset and linear regression models as the most appropriate methods for normalising the outputs of the co-located CO_2_ sensors, consistently with the study’s practical objectives. The chosen methods provide transparent, computationally efficient, and replicable approaches that are well established in the calibration of low-cost air quality sensors [15,28,38]. Simple bias adjustment enables correction of constant offsets between sensor and reference measurements, while linear regression accounts for both offset and proportional (gain) errors relative to the GR.

Bias Modelling:

Sensor bias (*B_i_*) for each sensor *i* was calculated as the mean difference between the sensor measurements and the corresponding GR measurements across all timestamps, as shown in Equation (1):

(1) $$B_{i}=\;\frac{1}{n}\sum_{t=1}^{n}\left(S_{i,t}-R_{t}\right)$$

where *S_i,t_* is the CO_2_ concentration measured by sensor *i* at time *t*, *R_t_* is the corresponding reference reading, and *n* is the total number of time points.

Each sensor’s measurements were then bias-corrected by subtracting the calculated bias value, as given in Equation (2):

(2) $${S^{\prime }}_{i,t}=\;S_{i,t}-\;B_{i}$$

Regression Modelling:

A simple linear regression model was also applied to each sensor to correct both offset and scaling deviations relative to the reference. The regression relationship between the sensor and reference measurements is described by Equation (3):

(3) $$R_{t}=\;a_{i}+\;b_{i}S_{i,t}$$

where *a_i_* and *b_i_* are the intercept and slope coefficients of the regression fit for sensor *i*, respectively.

To normalise the sensor measurements, the inverse of the regression model was applied to adjust each sensor output, as shown in Equation (4):

(4) $${S^{\prime }}_{i,t}=\;\frac{R_{t}-\;a_{i}}{b_{i}}$$

Validation of the bias and regression correction models was performed using a leave-one-period-out (LOPO) cross-validation approach. In this method, data from two complete CPs were used for model training, while the remaining period served as an independent test set. The process was repeated iteratively until each period had been used once as the test dataset. The LOPO method is particularly well suited to environmental time series data, where sensor behaviour, ambient conditions, and occupancy-driven patterns can vary systematically between measurement periods.

Unlike randomised cross-validation methods (e.g., k-fold), which may allow leakage between temporally adjacent samples and artificially inflate performance estimates [39], LOPO preserves the temporal and contextual integrity of each CP. This reduces overfitting and yields a more conservative and representative assessment of model accuracy [40].

By validating models across discrete and independent contexts, such as different seasons, classroom locations, or occupancy profiles, LOPO provides a more stringent and transferable evaluation of correction performance [41].

Following normalisation, performance metrics were calculated for each test period for both correction models. The final model selection was based on the relative improvement in these metrics across all validation periods. The model demonstrated the greatest reduction (mean, SD and range) in error (RMSE and MBE) and improvement in consistency (CV and SEE), resulting in the lowest percentage of measurements outside of the manufacturer’s stated accuracy, and was selected as the preferred normalisation approach for subsequent field deployment and analysis.

The preferred normalisation model was then retrained using the combined dataset encompassing all three CPs to generate the final correction coefficients. This approach maximised the available training data, thereby improving the robustness and generalisability of the correction parameters derived across a range of environmental and operational conditions.

Following application of the normalisation corrections to the full dataset, post-normalisation performance metrics were calculated to assess model effectiveness.

#### 2.3.4. Statistical Tests

Paired *t*-tests were conducted to compare pre- and post-normalisation sensor outputs across all CPs, determining whether the mean differences between paired observations were statistically significant.

Pearson correlation coefficients (r) were calculated to assess potential systematic relationships between sensor residuals and environmental variables, including air temperature, relative humidity, and global reference (GR) CO_2_ concentration.

An event-based analysis was conducted to test whether out-of-range measurements (defined as deviations exceeding ±30 ppm + 3% from the GR) occurred during periods of significantly higher CO_2_ rates of change compared with non-exceedance periods, using paired *t*-tests.

## 3. Results and Discussion

### 3.1. Performance Metrics Pre-Normalisation

**Figure 1:**
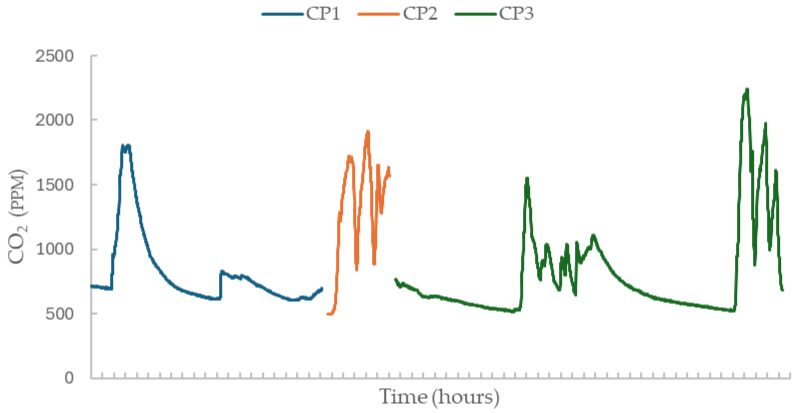
Temporal trend of GR CO_2_ across co-location periods.

Figure 1 shows the temporal trend of the GR across the three co-location periods, providing an overview of network-level CO_2_ behaviour against which the performance and accuracy of all 23 sensors were evaluated relative to the manufacturer’s specified accuracy of ±30 ppm + 3% [30]. Because low-cost NDIR sensors are subject to both random error and calibration drift [14], it is unrealistic to expect all measurements to remain strictly within the stated accuracy range over extended deployments and varying environmental conditions. Additionally, a small proportion of measurements may reasonably fall outside this range due to transient noise, environmental interferences, or short-term sensor response fluctuations [14,15].

Table 2 summarises the mean and SD of key performance metrics (RMSE, R^2^, MBE, CV, SEE), along with the proportion of measurements falling outside the manufacturers stated accuracy range across all CPs. Across the sensor network, 99.55% of CO_2_ measurements during the co-location tests remained within the manufacturers’ specified accuracy limits. The highest proportion of out-of-tolerance measurements observed for any individual sensor was 1.6% (Sensor 12), corresponding to an RMSE of 26.8 ppm across the three CPs.

The pre-normalisation CO_2_ performance metrics for each sensor, presented for each CP and for all periods combined, are provided in Tables S2–S5 of the Supplementary Materials. Overall, the network of co-located sensors demonstrated a strong consistency and high measurement accuracy in relation to the GR for CO_2_. RMSE averaged 18 ppm, ranging from 9 ppm (Sensor 7) to 31 ppm (Sensor 11), indicating a relatively low dispersion of sensor measurements relative to the GR. R^2^ values were exceptionally high (mean = 0.9986; range = 0.0024), suggesting an almost perfect linear agreement between the individual sensor outputs and the reference dataset. The wider range of MBE values, −26.8 ppm (Sensor 11) to 19.4 ppm (Sensor 5), reflects the influence of minor offset variations between sensors. The CV averaged 1.7% (range = 1.6%), further supporting strong precision and low variability among sensors. SEE averaged 13.3 ppm (range = 10.7 ppm), again indicating strong predictive accuracy relative to the reference. Only 0.45% of all measurements fell outside the manufacturer’s stated accuracy limits, confirming the high reliability and stability of the sensor network during co-location testing. Overall, these metrics confirm that the sensors exhibit acceptable agreement, minimal systematic bias, and high reproducibility.

The time series plots (Figures S1–S3 of the Supplementary Materials) illustrate the temporal agreement between sensors across each CP. The network of sensors demonstrated similar dynamic responses, tracking changes in indoor CO_2_ reasonably well throughout the monitoring periods. Divergence between sensors was smallest at lower concentrations, with differences between the highest- and lowest-reading sensors of ~50 ppm observed at measurements <600 ppm. However, as CO_2_ levels increased, the spread between the highest and lowest measurements widened, reaching up to ~150 ppm at elevated concentrations of ~1800 ppm. The scatter plots (Figure S4 of the Supplementary Materials) further support these observations. While most sensors exhibited strong linear relationships and followed consistent trend lines, a small number of outliers were identified, predominantly during CP1, likely attributable to intermittent proximity to occupant breath plumes. The proportional bias varied across the measurement range, with the difference between the highest- and lowest-reading sensors growing from 56 ppm (SD = 15) at measurements below 600 ppm to 131 ppm (SD = 35) at concentrations between 1750 ppm and 2000 ppm before reducing to 111 ppm (SD = 29) at concentrations between 2000 ppm and 2250 ppm.

Across the 23 co-located sensors, correlations between residuals and temperature, relative humidity, and global reference concentration were consistently small (mean |r| ≤ 0.02; median |r| ≤ 0.02), with no consistent directionality, indicating no systematic environmental or concentration-dependent bias within the observed indoor ranges. While a small number of sensors exhibited moderate correlations with temperature or concentration, these effects were isolated and not consistent across the network, indicating no systematic dependence at the group level.

#### Analysis of Out-of-Range CO_2_ Measurements

**Figure 2:**
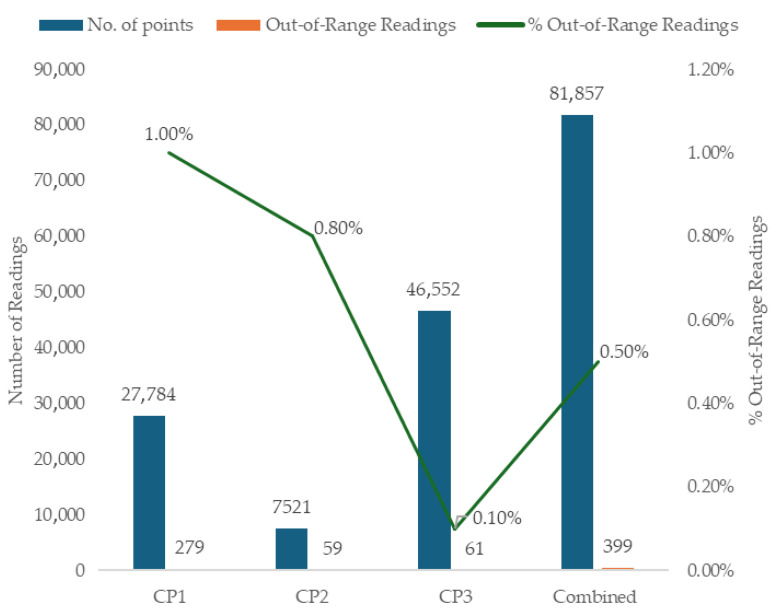
Percentage of out-of-range measurements for each co-location period and the combined dataset.

The proportion of CO_2_ measurements falling outside of the manufacturer’s stated accuracy limits varied substantially across CPs. As depicted in Figure 2, 70% of out-of-range measurements occurred during CP1, despite this period representing only 34% of the combined dataset. CP1 was conducted in a small office with sensors located in closer proximity to occupants than those in CP2 or CP3. The elevated proportion of out-of-range values in this period is likely attributable to localised breath plume effects and insufficient air mixing, conditions conducive to sharp short-term gradients in CO_2_ concentrations in the near-field of occupants. CP2 contributed 9% of the total dataset but accounted for 15% of out-of-range measurements. Although sensors were positioned along a well-mixed airflow pathway between the classroom windows and the door to the corridor, CP2 exhibited both higher mean CO_2_ concentrations and markedly greater temporal variability, with standard deviations 29% and 15% higher than those observed in CP1 and CP3, respectively. While the influence of occupants’ breathing cannot be discounted, the dynamic changes in indoor CO_2_ concentrations likely explain the comparatively higher proportion of out-of-range measurements (0.8%) relative to those in CP3. In contrast, the height and location of sensor positioning for CP3 negated the potential impact of occupant breath plumes and transient local peaks, resulting in a lower proportion (0.1%) of out-of-range measurements, thereby representing a more optimal sampling location for classroom-based co-location setups.

An event-based analysis was conducted to examine whether out-of-range measurements were associated with rapid short-term CO_2_ dynamics. Minute-to-minute rates of change were compared using paired, event-level analyses, showing that CO_2_ rates of change during out-of-range events were significantly higher (mean = 35 ppm min^−1^) than those during non-exceedance periods (mean = 4.5 ppm min^−1^; paired *t*-test, *p* < 0.001). These results support the interpretation that localised transient plumes and enhanced concentration dynamics, combined with inter-sensor response-time differences, contributed to the occurrence of out-of-range measurements.

**Figure 3:**
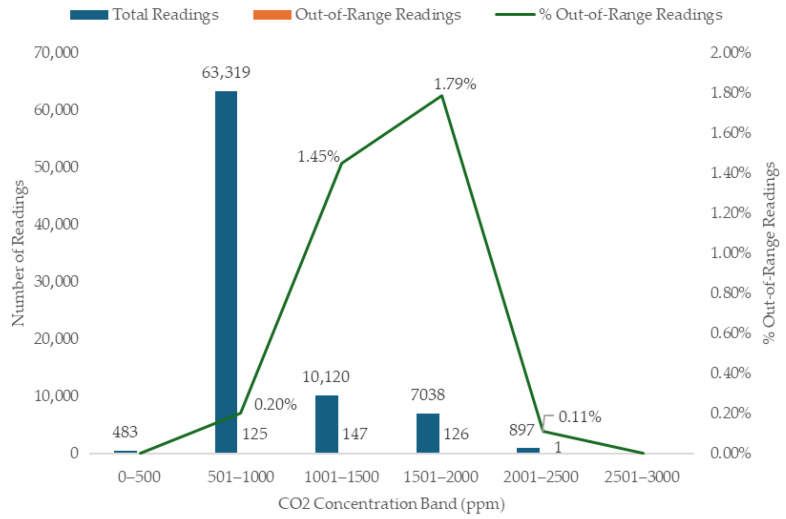
Distribution of sensor measurements exceeding accuracy limits across CO_2_ bands.

Figure 3 presents the distribution of sensor measurements that fell outside the manufacturer’s stated accuracy range across different bands of the GR CO_2_ concentration. The proportion of out-of-range values increased with rising CO_2_ concentration up to approximately 2000 ppm, confirming that sensor performance is influenced by concentration level.

At low concentrations (<500 ppm), no out-of-range measurements were recorded, reflecting high precision under background or near-outdoor conditions. In the 501 ppm to 1000 ppm range, which represents ideal occupied classroom conditions under adequate ventilation [3], the proportion of out-of-range measurements remained very low (0.20%). This suggests that the sensors perform robustly within the concentration range most relevant for practical ventilation assessment.

However, the percentage of out-of-range measurements increased noticeably at higher CO_2_ levels, associated with reduced ventilation or high occupancy. Specifically, 1.45% and 1.79% of measurements were out-of-range in the 1001 ppm to 1500 ppm and 1501 ppm to 2000 ppm bins, respectively. Interestingly, accuracy appeared to improve again in the 2001 ppm to 2500 ppm band (0.11% out-of-range measurements).

The minimal deviations observed at the lowest and highest concentration bands may be attributed to the relatively stable rates of change in CO_2_ concentrations typically occurring when classroom CO_2_ is at these levels. At lower concentrations (e.g., early morning pre-occupancy), classrooms had typically been unoccupied for a prolonged time, resulting in minimal CO_2_ variation, which reduced temporal sensitivity in the measurements. By contrast, the mid-range concentration bands (approximately 1000–2000 ppm) coincide with periods of active occupancy and fluctuating ventilation behaviour, when CO_2_ levels change more dynamically due to varying occupant density, intermittent window opening, and door movements.

For example, during the first 45 min of classroom occupancy in CP3, CO_2_ levels increased from 534 ppm to 1763 ppm, corresponding to a build-up rate of 27.3 ppm per minute. During these more rapid changes, even small temporal offsets associated with one-minute sampling intervals could introduce detectable discrepancies in the recorded concentration values. This increased temporal variability likely contributed to the higher proportion of out-of-range measurements observed in the mid-concentration bands, where CO_2_ levels were undergoing active growth or decay.

Similarly, during classroom break time in CP3, as occupants vacated the room and windows remained open, CO_2_ levels decayed from 2183 ppm to 1620 ppm over 30 min, corresponding to a decay rate of approximately 19 ppm per minute. This decay rate represents a relatively steep short-term concentration change, which may have resulted in temporal misalignment where minor response-curve differences between sensors became more pronounced.

Conversely, the slight reduction in out-of-range measurements observed again at very high concentrations may reflect periods in which classroom CO_2_ levels approach a quasi-steady state or, more likely, the end of the school day, when despite high absolute CO_2_ values, the rate of change shifts to a slow overnight decay. For example, at the end of the first school day in CP3, once the room was vacated, CO_2_ levels decayed from 1106 ppm to 749 ppm over 180 min, corresponding to a decay rate of approximately 2 ppm per minute. Thus, the pattern of out-of-range values appears to be driven not simply by absolute concentration but by the rate of concentration change, which influences the sensitivity of the analysis to sampling interval timing across the sensor network.

Overall, these results demonstrate that sensor accuracy is highest within the CO_2_ concentration range most relevant to classroom ventilation management (500 ppm to 1500 ppm). A modest decline in performance is observed at higher concentrations (>1500 ppm), but the magnitude of this effect is small in absolute terms (<2%). This has practical implications for VR estimation, while data within the lower-to-mid CO_2_ concentration range can be used with high confidence. However, VRs derived from periods of elevated or dynamic concentrations should be interpreted with caution or by averaging values over multiple time intervals to reduce uncertainty.

### 3.2. Normalisation Model Validation Results

The results of the pre- and post-normalisation model (bias and regression) performance metrics for each sensor are tabulated in Tables S6–S14 of the Supplementary Materials. Table 3 presents performance metrics for the sensors across three CPs: pre-adjustments, post-bias adjustments, and post-regression adjustments. The results demonstrate consistent improvements in accuracy and precision following the application of bias and regression corrections, with the strongest performance observed in CP3.

Overall, bias adjustment effectively reduced systematic offsets, while regression adjustment maintained linearity while reducing proportional errors, particularly at higher CO_2_ concentrations. Post-adjustment results show a clear improvement in RMSE across all co-location periods. In CP1, RMSE was reduced by 6% using bias correction and by 4% using regression. More substantial improvements were observed in CP2, with reductions of 24% and 25% for bias and regression adjustments, respectively. CP3 also showed notable improvements, with RMSE reductions of 15% following bias correction and 21% following regression adjustment, demonstrating the effectiveness of both approaches, particularly under more variable conditions. To account for the differing number of data points across CPs, weighted averages were calculated for both bias and regression performance metrics. When weighted by the number of observations, the overall percentage improvement in RSME across all CPs was 12.6% for bias correction and 16.0% for regression.

Table 4 summarises the weighted mean, minimum and maximum performance metrics across all CPs for the pre-adjusted sensor data and for the bias and regression-adjusted datasets. Overall, the results demonstrate that both adjustment approaches lead to modest but consistent improvements in accuracy across the indoor CO_2_ concentration range typically observed in NV classrooms.

It is important to note that the magnitude of these improvements must be interpreted in the context of the relatively high accuracy and stability of the sensors prior to adjustment. The pre-correction performance already demonstrated low RMSE values and strong temporal agreement with the GR, which is broadly consistent with the performance reported for Aranet4 NDIR CO_2_ sensors [35]. Consequently, the scope for large performance gains through post-processing was inherently limited. If the baseline agreement had been weaker, for example, in sensors subject to greater drift or poorer optical stability, then the relative benefit of bias or regression correction would likely have been more pronounced, as reported by Dubey et al. [15]. In this sense, the modest improvements observed here should be viewed as refinements rather than corrections of major systematic errors. They demonstrate that the sensors were already tracking reference CO_2_ concentrations with high fidelity and that the calibration procedures primarily served to reduce small but systematic offsets, improving confidence in derived VR estimates without fundamentally altering the underlying measurement behaviour.

RMSE decreased from a weighted mean of 18.1 ppm in the pre-adjustment dataset to 15.7 ppm following bias correction and 15.1 ppm after regression adjustment. While the absolute magnitude of improvement is relatively small (a reduction of ~3 ppm), these reductions are consistent across the range of concentrations. The maximum RMSE also declined substantially, from 38.6 ppm pre-adjustment to 34.6 ppm with bias correction and 33.2 ppm with regression, indicating that the adjustments are especially effective during periods with higher indoor CO_2_ variability (e.g., high occupancy or limited ventilation).

R^2^ values remained extremely high across all datasets (weighted mean = 0.9987), with negligible differences across the adjustment approaches. This indicates that the raw sensors already tracked temporal variation in reference concentration well and that calibration primarily affects offset and proportional bias rather than signal responsiveness.

The MBE results require careful interpretation. The “minimum” values reported (e.g., −35.0 ppm pre-adjustment) reflect the direction of mean bias rather than absolute error. The weighted mean MBE increased slightly with bias and regression adjustment (from 0.1 ppm to ~0.4 ppm), which is expected because the adjustments prioritised reducing systematic offset rather than minimising absolute deviation across all points. Importantly, both adjustment methods reduced systematic bias, as reflected in the reduced RMSE and balanced error distribution.

The CV remained low (<2% on average) in all cases, highlighting the stability and consistency of the sensor response. Slight reductions were observed following bias correction (1.59% to 1.53%), though regression adjustment did not reduce the CV further. SEE showed minimal variation across adjustment methods, with weighted mean values of 11.3 ppm to 11.4 ppm, indicating that the residual spread of differences after model fitting is broadly similar across calibration approaches.

Finally, the percentage of measurements outside the manufacturer’s stated accuracy range decreased following adjustment, from 0.45% pre-adjustment to 0.37% (bias) and 0.39% (regression). The maximum deviation also reduced from 0.92% to 0.84% (bias) and 0.83% (regression), indicating that calibration improves reliability particularly in the upper tail of the error distribution.

Given the high baseline tracking fidelity (R^2^ > 0.996 in all cases), the primary source of error arises from systematic offset rather than dynamic instability. This aligns with known drift behaviour in NDIR sensors and supports the use of periodic short-term co-location calibrations to maintain data quality in long-term deployments.

### 3.3. Performance Metrics Post-Normalisation

Regression-based correction was selected for normalisation of sensor outputs on the basis that it led to marginally greater improvements in precision metrics in the validation models. Regression coefficients were derived from all available co-location data to ensure robustness across seasonal and operational variability. To statistically verify the effect of normalisation, a paired *t*-test was performed, comparing pre- and post-normalisation sensor outputs across all three CPs. The result (*p* < 0.001) demonstrates that mean differences between paired observations were statistically significant following regression correction. The mean reduction in RMSE was 5 ppm, representing a 27% decrease in overall error, accompanied by a very strong correlation between pre- and post-normalisation paired values (r = 0.999 ± 0.001). The maximum percentage of measurements outside of stated accuracy for a single sensor fell by 62.5% from 1.6% (Sensor 12) pre-normalisation to 0.6% (Sensor 5 and Sensor 12) post-normalisation. Furthermore, the proportion of measurements falling outside the manufacturer’s stated accuracy range across the sensor network decreased by 43% (from 0.45% to 0.26%), reflecting that regression correction primarily introduced a systematic alignment shift rather than altering the fundamental response behaviour of the sensors.

A summary of the mean, SD, maximum and minimum performance metrics before and after normalisation is presented in Table 5, while per-sensor regression coefficients and detailed pre/post-precision metrics are provided in Table S15 of the Supplementary Materials.

Time series examination of the post-normalisation data (Figures S5–S7 of the Supplementary Materials) indicates reduced divergence between sensors across the range of observed CO_2_ levels. Extreme differences between sensors were typically ~30 ppm at concentrations near 600 ppm. As CO_2_ levels increased, the spread between the highest and lowest sensor measurements widened, reaching ~115 ppm at elevated concentrations around 1800 ppm. The post-normalisation scatter plots (Figure S8 of the Supplementary Materials) show a clear reduction in proportional bias across the measurement range, with the difference between the highest- and lowest-reading sensors decreasing by an average of 31% across the dataset. The SD between individual sensors and the GR also decreased by an average of 29% (from 19 ppm to 14 ppm) across the full dataset. However, the pattern of proportional bias remained broadly consistent with pre-normalisation behaviour, with a SD of 8 ppm at concentrations below 600 ppm, increasing to 27 ppm between 1750 and 2000 ppm, before declining to 20 ppm at concentrations between 2000 and 2250 ppm.

These results indicate that normalisation effectively corrects systematic offsets, but residual proportional divergence persists at higher and more dynamic CO_2_ levels, consistently with known scaling effects of low-cost NDIR sensors. The persistence of proportional divergence at higher CO_2_ concentrations has important implications for the interpretation of sensor-based IAQ and ventilation analyses. Elevated concentration ranges typically coincide with more dynamic occupancy patterns and less stable mixing conditions, potentially amplifying the influence of sensor time synchronisation. As a result, ventilation metrics derived during peak periods of CO_2_ rates of change may exhibit greater uncertainty even after normalisation.

The practical implication of these results is that post-normalisation data are more reliable for comparing spatial variances in CO_2_ with multiple sensors and for estimating VRs, particularly during periods of moderate indoor CO_2_ levels (e.g., typical classroom occupancy conditions). The remaining divergence at higher concentrations suggests that VR estimates derived from steep decay curves or from conditions approaching 2000 ppm should be interpreted with caution or averaged across multiple sensors when feasible. However, the substantial reduction in systematic offset and out-of-spec measurements provides increased confidence in using these sensors for long-term monitoring and comparative analysis across classrooms.

### 3.4. Precision of Sensor Measurements Across CO_2_ Threshold Ranges

The precision of the pre- and post-normalised CO_2_ measurements was further evaluated across three threshold ranges (<600 ppm, <1000 ppm, and <1500 ppm), representing ambient conditions and levels commonly used as indoor air quality decision points [7]. The results, presented in Table 6, confirm that sensor precision within these ranges is substantially higher than when assessed across the full concentration span of the dataset (494–2244 ppm). Post-normalisation, RMSE values decreased consistently across all thresholds, with the most pronounced improvement observed in the <600 ppm range (from 13.5 ± 6.3 ppm to 7.4 ± 1.5 ppm). Similar reductions were seen at <1000 ppm (15.1 ± 5.2 ppm to 10.4 ± 2.3 ppm) and <1500 ppm (16.7 ± 5.1 ppm to 11.9 ± 2.4 ppm), further emphasising the enhanced agreement between individual sensors and the GR at both ambient and moderate CO_2_ concentrations. At ambient levels, no measurements were recorded outside of manufacturer’s stated accuracy after correction, this number increased marginally at the higher reference thresholds.

## 4. Limitations

This study evaluated sensors from a single manufacturer, which may limit the generalisability of the findings to other low-cost CO_2_ sensor types. The co-location period was relatively short (six months), constraining the assessment of longer-term performance and drift under a broader range of environmental conditions. Pre-correction sensor performance was already strong, characterised by low RMSE values and high temporal agreement with the global reference (GR); consequently, the scope for further improvement through post-processing was inherently limited. Some observed discrepancies may also reflect short-term temporal variability associated with one-minute sampling intervals, and the influence of localised occupant breath plumes cannot be entirely excluded.

Potential cross-sensitivities to other indoor gases and environmental factors, such as relative humidity, were not explicitly isolated. However, as all sensors were co-located and operated within the same classroom microenvironment, any such interference effects were shared across devices, supporting the use of on-site co-location as a practical means of reducing inter-sensor variability under typical classroom conditions. Relatedly, findings regarding temperature and humidity dependence are restricted to group-level observations, as individual sensor responses could not be differentiated under identical exposure conditions.

The absence of an independent reference-grade CO_2_ instrument represents a departure from conventional performance evaluations that prioritise absolute accuracy against a traceable standard (e.g., laboratory calibration or field co-location with a reference instrument). Accordingly, the conclusions are intentionally limited to relative normalisation, inter-sensor agreement, and ΔCO_2_ dynamics and do not extend to claims of absolute accuracy against a reference-grade standard.

To maintain methodological transparency and accessibility, only mean bias correction and simple linear regression were applied. More complex non-linear or machine learning-based calibration approaches were not considered; therefore, the findings are limited to linear normalisation methods.

## 5. Conclusions and Recommendations

Networked, low-cost NDIR CO_2_ sensors offer a practical and scalable solution for monitoring and assessing VRs in schools and other indoor environments. VR calculations depend on the I/O ΔCO_2_ rather than absolute concentrations. Therefore, a network-based GR offers a dependable alternative to calibration with certified reference instruments. It allows for reliable, comparable measurements across sensor networks while avoiding the cost and logistical burden of accessing specialised calibration equipment. Furthermore, co-location within the deployment environment proves a robust method for deriving correction factors, particularly when test periods capture the full CO_2_ cycle and avoid localised occupant effects.

This study demonstrates that low-cost NDIR CO_2_ sensors, when appropriately normalised, can provide highly comparable measurements suitable for classroom ventilation assessment. While pre-normalisation measurements showed marginal inter-sensor variability, correction models significantly improved consistency, reducing RMSE by 27% (from 18.3 ppm to 13.4 ppm), mean proportional bias by 31% (from 68 ppm to 46 ppm), and out-of-range values by 43% (from 0.45% to 0.26%) across the full dataset.

Overall, the findings confirm that low-cost NDIR CO_2_ sensors, when appropriately normalised, can provide highly comparable measurements suitable for evaluating classroom ventilation. While pre-normalisation measurements showed modest inter-sensor variability, the application of correction models substantially improved performance, reducing RMSE by 27% (from 18.3 ppm to 13.4 ppm), mean proportional bias by 31% (from 68 ppm to 46 ppm), and out-of-range values by 43% (from 0.45% to 0.26%) across the full dataset.

The improvement is particularly evident at key threshold levels used for classroom ventilation assessment (≤600 ppm, ≤1000 ppm, and ≤1500 ppm). At these thresholds, post-normalisation RMSE values improved by 45% (13.5 ppm to 7.4 ppm), 31% (15.1 ppm to 10.4 ppm), and 29% (16.7 ppm to 11.9 ppm), respectively.

Slight performance declines were observed during periods of dynamic and elevated CO_2_ concentrations. Across the pre-normalisation dataset, 1.79% of measurements in the 1501 ppm to 2000 ppm range were out-of-range, indicating that VRs derived under these conditions should be interpreted with caution or averaged across multiple intervals to reduce uncertainty.

While laboratory-based testing across the full range of CO_2_, temperature, and relative humidity conditions would offer a more comprehensive assessment of sensor performance, such evaluations are generally impractical for most low-cost sensor users, including schools and other resource-constrained buildings. Given these limitations, the development of practical, user-friendly field calibration guidelines for low-cost NDIR sensors is highly valuable. Additionally, a manufacturer-supported application that allows users to conduct structured co-location procedures under real deployment conditions (with or without reference instruments) would significantly enhance data reliability. By automatically deriving and applying correction functions across sensor networks, such an application would greatly improve the quality of ventilation assessments. Future research conducted in deployment conditions could incorporate high-precision reference instruments and explore advanced machine learning approaches to compare these findings and further improve calibration accuracy and long-term stability.

### Recommendations for Field Co-Location Deployment

The following recommendations are outlined in relation to the deployment of field co-location for CO_2_ sensors:

- Sensors should be placed side by side in a representative deployment location with adequate air mixing and away from potential interferences, such as occupant breath plumes (e.g., at a height of 2.2 m).
- Sensors should be exposed to a full range of representative measurements by including both occupied and unoccupied periods for at least one full cycle (24 h).
- Periods of unstable mixing patterns or dynamic occupancy patterns leading to rapid CO_2_ build-up or decay should be treated cautiously or excluded, as they can amplify the influence of sensor time synchronisation and potentially bias results.
- Simple correction models can effectively adjust measurements, with regression offering marginally better performance than bias correction.
- Co-location calibrations should be repeated regularly and include all seasons to capture the variations in temperature and RH during deployment.

## Figures and Tables

**Table 1:** Range of internal environmental parameters recorded during each co-location period (CP) between January and June 2025.

		CP1 (19–20 January ‘25)	CP2 (10 March ‘25)	CP3 (10–12 June ‘25)	Combined Dataset
**CO_2_** **(ppm)**	Mean	810	1309	795	847
	Median	705	1427	651	700
	SD	286	406	347	365
	Maximum	1805	1915	2244	2244
	Minimum	603	494	516	494
**Temperature** **(°C)**	Mean	19.4	17.8	18.5	18.7
	Median	20.1	18.3	18.3	18.7
	SD	2.2	1.9	0.7	1.6
	Maximum	20.5	19.9	20.6	20.6
	Minimum	12.9	14.5	17.7	12.9
**RH** **(%)**	Mean	58	62	70	62
	Median	55	62	70	62
	SD	5	2	2	6
	Maximum	77	65	77	77
	Minimum	54	59	67	54
**Pressure** **(hPa)**	Mean	1014	1007	1013	1013
	Median	1014	1007	1014	1014
	SD	0.6	0.5	4.8	4.1
	Maximum	1015	1007	1019	1019
	Minimum	1013	1005	1005	1005

**Table 2:** Summary of mean and standard deviation of pre-normalisation sensor performance metrics (RMSE, R^2^, MBE, CV, SEE) and the proportion of measurements exceeding the manufacturer-specified accuracy limits across all co-location periods (CPs).

RMSE	R^2^	MBE	CV (%)	SEE	Out-of-Range Measurements (%)
18.335 ± 5.322	0.999 ± 0.001	0.098 ± 13.091	1.674 ± 0.345	13.332 ± 2.511	0.45%

**Table 3:** Mean and SD values for pre- and post-adjustment performance metrics.

Precision Metric	Pre-Adjustments			Post-Bias Adjustment			Post-Regression Adjustment		
	Co-Lo 1	Co-Lo 2	Co-Lo 3	Co-Lo 1	Co-Lo 2	Co-Lo 3	Co-Lo 1	Co-Lo 2	Co-Lo 3
RMSE (ppm)	18.8 ± 5.5	21.9 ± 6.8	17.0 ± 6.3	17.7 ± 5.4	16.7 ± 4.9	14.4 ± 5.5	17.9 ± 5.5	16.4 ± 3.7	13.3 ± 5.7
R^2^	0.9979 ± 0.0007	0.9987 ± 0.0006	0.9992 ± 0.0002	0.9979 ± 0.0007	0.9987 ± 0.0006	0.9992 ± 0.0002	0.9979 ± 0.0007	0.9987 ± 0.0006	0.9992 ± 0.0002
MBE (ppm)	−0.6 ± 11.6	−0.2 ± 17.5	0.6 ± 15.0	−1.4 ± 9.6	−0.5 ± 8.6	1.6 ± 11.5	−1.6 ± 9.7	−0.1 ± 7.3	1.7 ± 9.4
CV (%)	2.0 ± 0.4	1.2 ± 0.2	1.4 ± 0.2	2.0 ± 0.4	1.2 ± 0.2	1.3 ± 0.2	2.0 ± 0.4	1.2 ± 0.2	1.4 ± 0.2
SEE (ppm)	12.9 ± 2.3	14.5 ± 2.9	9.9 ± 1.4	12.9 ± 2.3	14.5 ± 2.9	9.9 ± 1.4	12.9 ± 2.3	14.6 ± 2.9	9.9 ±1.4
% Outside Stated Accuracy	0.9%	0.7%	0.1%	0.8%	0.3%	0.1%	0.8%	0.3%	0.1%

**Table 4:** Weighted mean, max and min values for pre- and post-adjustment performance metrics (all co-location periods).

Precision Metric	Average			Max			Min		
	Pre	Bias	Regression	Pre	Bias	Regression	Pre	Bias	Regression
RMSE (ppm)	18.1	15.7	15.1	38.6	34.6	33.2	9.0	9.1	8.2
R^2^	0.999	0.999	0.999	0.999	0.999	0.999	0.997	0.997	0.997
MBE (ppm)	0.1	0.4	0.4	28.2	29.2	29.4	−35.0	−32.1	−31.9
CV (%)	1.59	1.53	1.59	2.85	2.85	2.87	0.80	0.85	0.85
SEE (ppm)	11.3	11.3	11.4	23.1	23.1	23.1	7.5	7.5	7.6
% Outside Stated Accuracy	0.45%	0.37%	0.39%	0.92%	0.84%	0.83%	0.12%	0.10%	0.13%

**Table 5:** Summary of performance metrics (mean, SD max and min) before and after normalisation.

Precision Metric	Average		SD		Max		Min	
	Pre	Post	Pre	Post	Pre	Post	Pre	Post
RMSE (ppm)	18.34	13.35	5.32	2.51	30.53	20.21	9.49	9.49
R^2^	0.999	0.999	<0.001	<0.001	0.999	0.999	0.997	0.997
MBE (ppm)	0.10	−0.07	13.09	0.0000	19.39	−0.07	−26.80	−0.07
CV (%)	1.67	1.60	0.3453	0.3147	2.73	2.47	1.11	1.12
SEE (ppm)	13.33	13.35	2.5109	2.5060	20.20	20.18	9.47	9.49

**Table 6:** Precision metrics pre- and post-normalisation across key CO_2_ concentration thresholds.

Metric	<600 ppm		<1000 ppm		<1500 ppm	
	Pre	Post	Pre	Post	Pre	Post
RMSE (ppm)	13.5 ± 6.3	7.4 ± 1.5	15.1 ± 5.2	10.4 ± 2.3	16.7 ± 5.1	11.9 ± 2.4
R^2^	0.928 ± 0.017	0.927 ± 0.017	0.992 ± 0.004	0.992 ± 0.004	0.997 ± 0.001	0.997 ± 0.001
MBE (ppm)	0.9 ± 13.4	0.1 ± 2.8	0.2 ± 12.3	−0.1 ± 0.2	0.2 ± 12.6	−0.1 ± 0.2
CV (%)	1.3 ± 0.2	1.3 ± 0.2	1.6 ± 0.3	1.5 ± 0.4	1.6 ± 0.3	1.6 ± 0.3
SEE (ppm)	6.6 ± 0.7	6.6 ± 0.7	10.2 ± 2.2	10.3 ± 2.2	11.8 ± 2.3	11.9 ± 2.3
% Outside Stated Accuracy	0.01%	0.00%	0.18%	0.14%	0.34%	0.20%

## Data Availability

The original contributions presented in this study are included in the article/Supplementary Materials. Further inquiries can be directed to the corresponding author.

## Supplementary Materials

The following supporting information can be downloaded at https://www.mdpi.com/article/10.3390/s26041265/s1, Figure S1: Time series plot of CO_2_ levels in CP1; Figure S2: Time series plot of CO_2_ levels in CP2; Figure S3: Time series plot of CO_2_ levels in CP3; Figure S4: Scatter plot of individual sensor measurements versus the global reference; Figure S5: Post-normalisation time series plot of CO_2_ levels across CP1; Figure S6: Post-normalisation time series plot of CO_2_ levels across CP2; Figure S7: Post-normalisation time series plot of CO_2_ levels across CP3; Figure S8: Post-normalisation scatter plot of individual sensor measurements versus the global reference; Table S1: Performance metrics for CO_2_ sensor evaluation; Tables S2–S5. Performance metrics pre-normalisation for each CO_2_ sensor for each co-location period and the combined dataset; Tables S6–S14: Results of pre- and post-normalisation performance metrics for each sensor for bias and regression models applied to each co-location period; Table S15: Regression coefficients and performance metrics for each sensor pre- and post-normalisation based on combined dataset.
