# Supplementary material for: Low-Cost CO2 Sensors: On-Site Performance Evaluation and Co-Location Correction Procedure for Reliable Ventilation Assessments in Schools
Source: Sensors (Basel). 2026 Feb 15;26(4):1265. doi: 10.3390/s26041265 (PMC12943829; doi:10.3390/s26041265)
Supplement: Supplementary file 1 [file sensors-26-01265-s001.zip › sensors-4124987-supplementary.pdf]

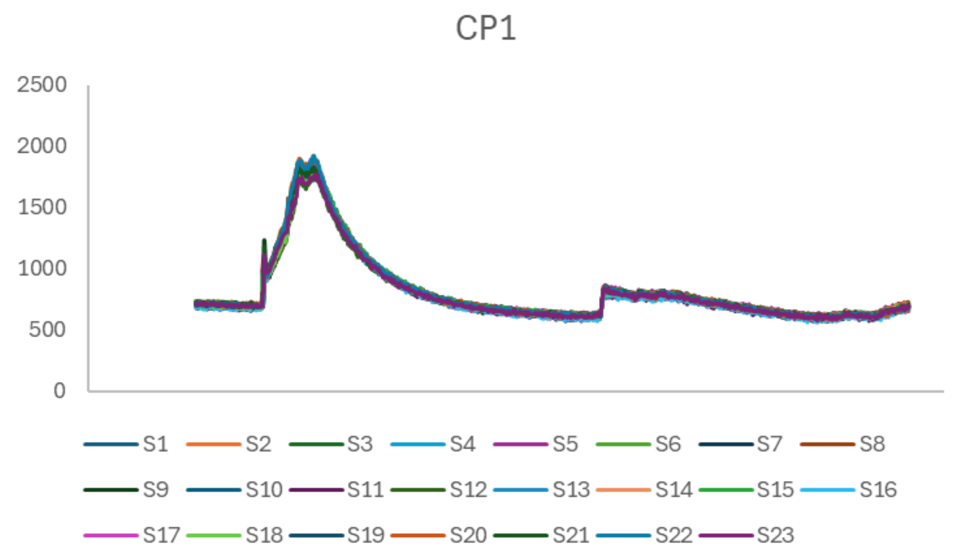

Figure S1. Time series plot of CO2 levels in CP1

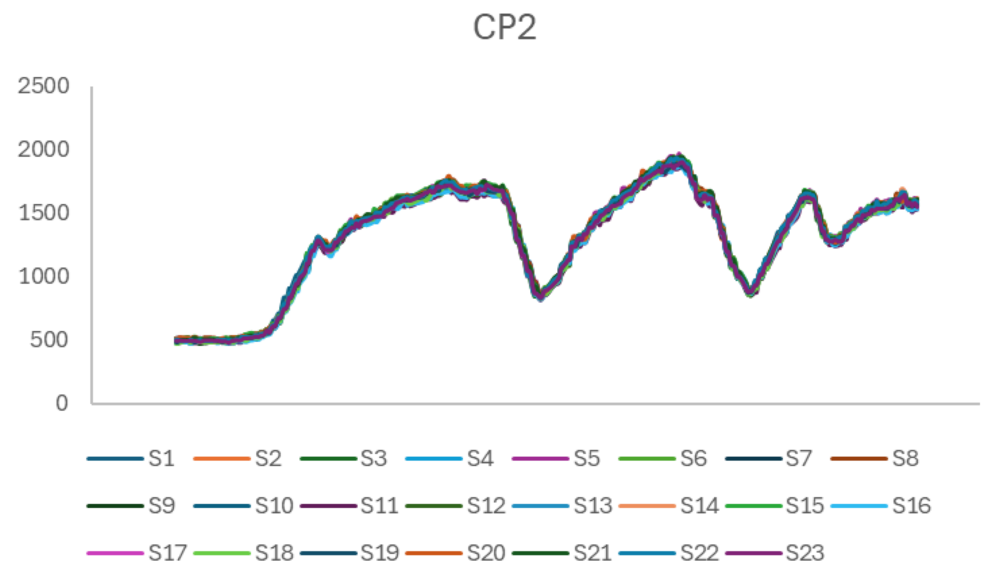

Figure S2. Time series plot of CO2 levels in CP2

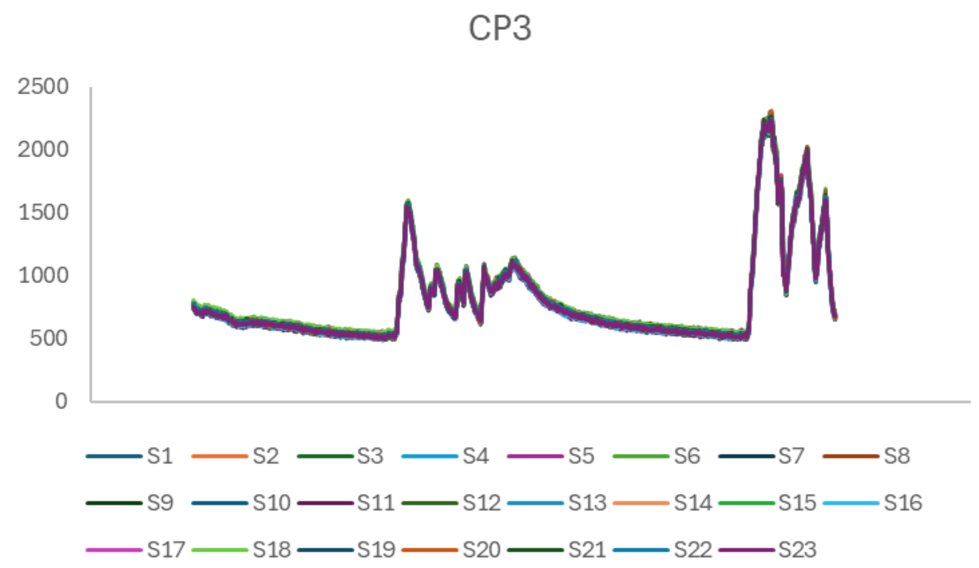

Figure S3. Time series plot of CO2 levels in CP3

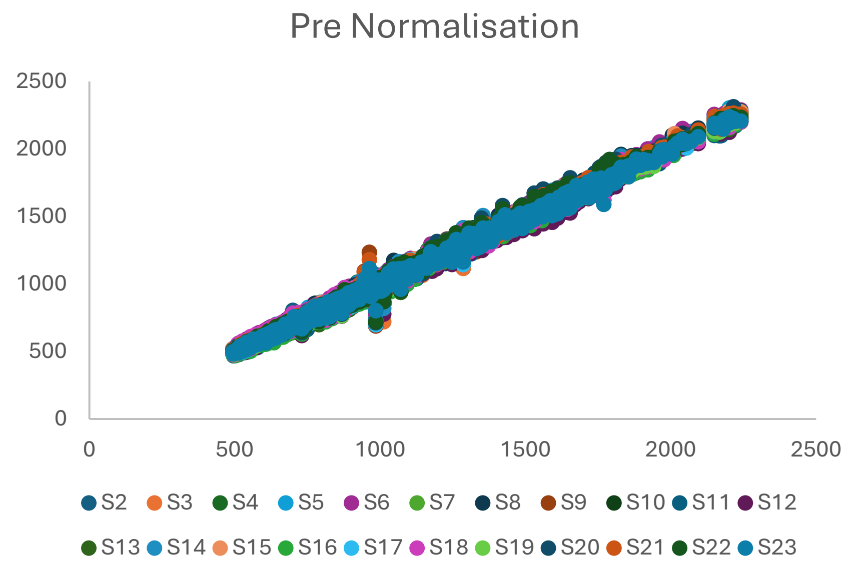

Figure S4. Scatter plot of individual sensor measurements versus the global reference

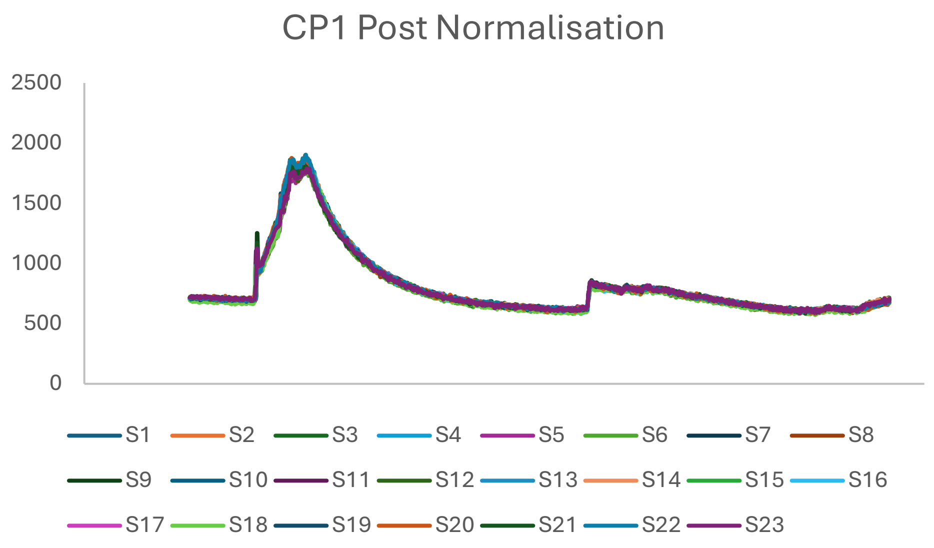

Figure S5. Post-normalisation time series plot of CO2 levels across CP1

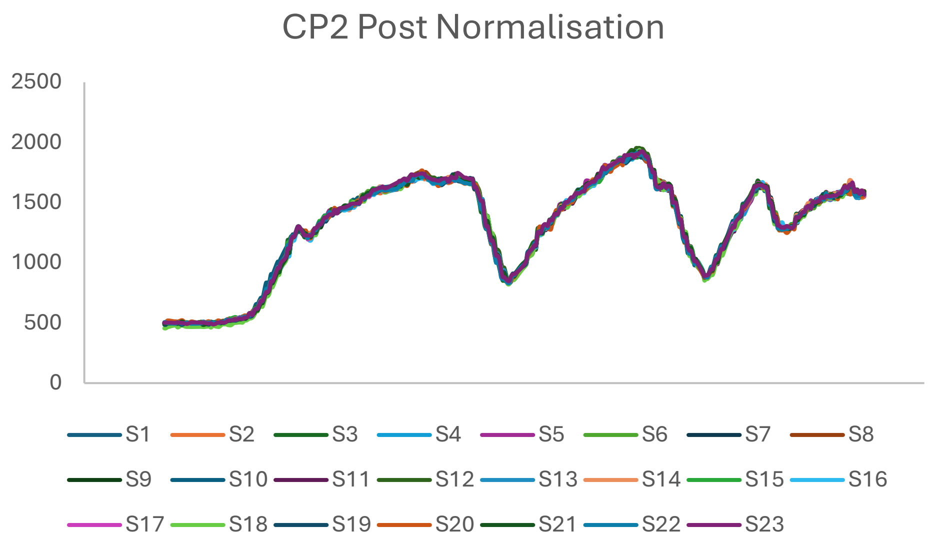

Figure S6. Post-normalisation time series plot of CO2 levels across CP2

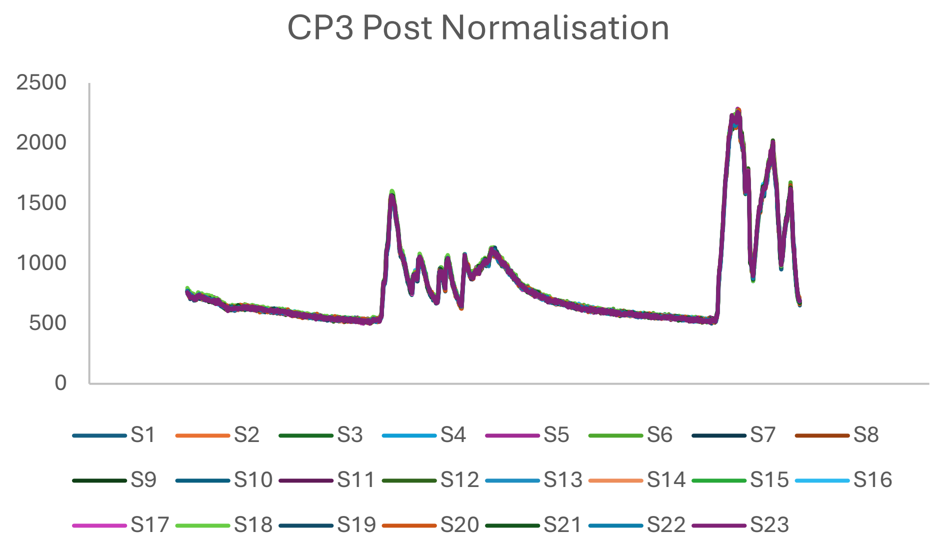

Figure S7. Post-normalisation time series plot of CO2 levels across CP3

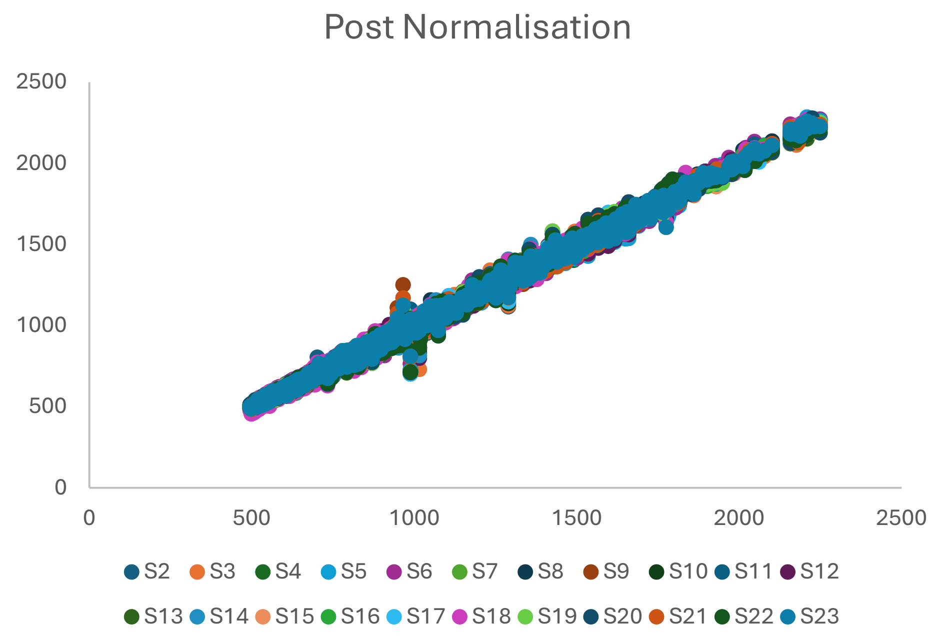

Figure S8. Post-normalisation scatter plot of individual sensor measurements versus the global reference

Table S1. statistical performance metrics

Metric	Formula	Interpretation	Performance Indicator
RMSE	$RMSE = \sqrt{\frac{1}{n}\sum(x_i - y_i)^2}$	Indicates the average magnitude of error between sensor and reference.	Lower values denote higher accuracy.
MBE	$MBE = \frac{1}{n}\sum(x_i - y_i)$	Represents mean bias: shows direction of deviation (positive = overestimation, negative = underestimation).	≈ 0 indicates minimal bias.
R^2	$R^2 = 1 - \frac{\sum(x_i - y_i)^2}{\sum(y_i - \bar{y})^2}$	Proportion of variance in reference data explained by sensor data (linearity).	→ 1 indicates strong linear correlation.
SEE	$SEE = \sqrt{\frac{\sum(x_i - \hat{y}_i)^2}{n-2}}$	Reflects the dispersion of data points around the regression line.	Lower SEE denotes better fit.
CV (%)	$CV = \frac{\sigma}{\bar{x}} \times 100$	Normalised measure of variability among sensors.	Lower CV% = greater sensor consistency.

Table S2. Colocation period 1. Performance metrics pre-normalisation for 23 co-located CO_2 sensors at 1-minute intervals

Sensor No.	RMSE (ppm)	R^2	MBE (ppm)	CV (%)	SEE (ppm)	<-30 ppm - 3%	>+30 ppm + 3%
S1	13	0.9981	3	1.6	12	0%	0%
S2	17	0.9982	1	2.1	12	0%	0%
S3	17	0.9975	-4	2.1	14	0%	1%
S4	18	0.9986	13	1.4	11	0%	0%
S5	23	0.9979	11	2.6	13	1%	0%
S6	13	0.9989	-5	1.6	9	0%	0%
S7	9	0.9990	1	1.1	9	0%	0%
S8	19	0.9983	13	1.8	12	0%	0%
S9	21	0.9965	-14	2.3	17	0%	0%
S10	14	0.9984	6	1.6	11	0%	0%
S11	32	0.9969	-27	2.3	16	3%	0%
S12	31	0.9975	-22	2.9	14	5%	0%
S13	13	0.9989	-3	1.6	9	0%	0%
S14	14	0.9984	5	1.6	11	0%	0%
S15	20	0.9987	15	1.6	10	0%	0%
S16	24	0.9981	-20	1.8	13	1%	0%

S17	16	0.9974	1	2.0	15	0%	1%
S18	17	0.9968	-5	2.2	16	1%	0%
S19	15	0.9977	-1	1.9	14	0%	1%
S20	24	0.9973	7	2.8	15	0%	4%
S21	17	0.9975	8	1.9	14	0%	0%
S22	22	0.9982	11	2.3	12	0%	4%
S23	21	0.9975	-9	2.5	14	1%	0%

Table S3. Colocation period 2. Performance metrics pre-normalisation for 23 co-located CO_2 sensors at 1-minute intervals

Sensor No.	RMSE (ppm)	R^2	MBE (ppm)	CV (%)	SEE (ppm)	<-30 ppm - 3%	>+30 ppm + 3%
S1	15	0.9987	-1	1.1	14	0%	0%
S2	15	0.9987	-6	1.0	14	0%	0%
S3	24	0.9968	-6	1.6	23	1%	1%
S4	23	0.9982	15	1.5	17	0%	2%
S5	29	0.9988	25	1.2	14	0%	3%
S6	22	0.9988	17	1.1	14	0%	1%
S7	13	0.9990	1	1.0	13	0%	0%
S8	25	0.9989	21	1.2	14	0%	1%
S9	24	0.9992	-22	1.0	11	0%	0%
S10	24	0.9977	14	1.4	19	0%	2%
S11	39	0.9987	-35	1.4	14	2%	0%
S12	26	0.9984	-20	1.5	16	1%	0%
S13	16	0.9991	-11	1.1	12	0%	0%
S14	18	0.9989	12	1.1	13	0%	1%
S15	28	0.9989	24	1.2	14	0%	1%
S16	34	0.9988	-31	1.2	14	1%	0%
S17	13	0.9992	-6	0.8	11	0%	0%
S18	25	0.9980	-18	1.5	18	1%	0%
S19	15	0.9991	-9	1.0	12	0%	0%
S20	21	0.9983	12	1.4	17	0%	1%
S21	20	0.9993	15	1.0	11	0%	0%
S22	18	0.9985	9	1.2	16	0%	0%
S23	14	0.9991	-7	0.9	12	0%	0%

Table S4. Colocation period 3. Performance metrics pre-normalisation for 23 co-located CO_2 sensors at 1-minute intervals

Sensor No.	RMSE (ppm)	R^2	MBE (ppm)	CV (%)	SEE (ppm)	<-30 ppm - 3%	>+30 ppm + 3%
S1	14	0.9987	-6	1.6	13	0%	0%
S2	10	0.9992	2	1.2	10	0%	0%
S3	16	0.9992	-12	1.3	10	0%	0%
S4	16	0.9995	14	1.1	8	0%	0%
S5	25	0.9993	23	1.4	9	0%	0%
S6	30	0.9991	28	1.5	10	0%	0%
S7	9	0.9993	-1	1.1	9	0%	0%
S8	21	0.9991	18	1.5	11	0%	0%
S9	19	0.9995	-16	1.1	8	0%	0%
S10	12	0.9992	7	1.3	10	0%	0%
S11	28	0.9993	-25	1.5	9	0%	0%
S12	24	0.9994	-22	1.2	9	0%	0%
S13	16	0.9993	-13	1.3	9	0%	0%
S14	12	0.9990	4	1.5	11	0%	0%
S15	17	0.9994	14	1.3	9	0%	0%
S16	22	0.9991	-19	1.5	10	0%	0%
S17	13	0.9993	-8	1.2	9	0%	0%
S18	27	0.9984	22	2.1	14	0%	0%
S19	12	0.9991	-6	1.3	10	0%	0%
S20	11	0.9991	3	1.4	10	0%	0%
S21	13	0.9993	9	1.3	9	0%	0%
S22	10	0.9992	3	1.3	10	0%	0%
S23	13	0.9991	-8	1.3	10	0%	0%

Table S5. Combined Dataset (all Colocation Periods). Performance metrics pre-normalisation for 23 co-located CO_2 sensors at 1-minute intervals

Sensor No.	RMSE (ppm)	R^2	MBE (ppm)	CV (%)	SEE (ppm)	<-30 ppm - 3%	>+30 ppm + 3%
S1	14	0.9986	-2	1.59	14	0%	0%
S2	13	0.9988	1	1.53	13	0%	0%
S3	17	0.9984	-9	1.72	15	0%	0%
S4	17	0.9992	14	1.27	10	0%	0%

S5	25	0.9981	19	1.96	16	0%	0%
S6	25	0.9973	16	2.34	19	0%	0%
S7	9	0.9993	0	1.11	9	0%	0%
S8	21	0.9988	17	1.60	13	0%	0%
S9	20	0.9988	-16	1.57	12	0%	0%
S10	14	0.9990	7	1.44	12	0%	0%
S11	31	0.9988	-27	1.82	13	2%	0%
S12	27	0.9984	-22	1.92	14	2%	0%
S13	15	0.9989	-9	1.46	12	0%	0%
S14	14	0.9988	5	1.51	13	0%	0%
S15	19	0.9992	15	1.41	10	0%	0%
S16	24	0.9988	-20	1.61	13	0%	0%
S17	14	0.9987	-5	1.53	13	0%	0%
S18	24	0.9969	9	2.73	20	1%	0%
S19	13	0.9988	-4	1.50	13	0%	0%
S20	17	0.9984	5	1.95	15	0%	1%
S21	15	0.9990	9	1.49	12	0%	0%
S22	16	0.9986	6	1.72	14	0%	0%
S23	16	0.9986	-8	1.72	14	0%	0%

Table S6. Co-location period 1. Performance metrics pre-normalisation for 23 co-located CO_2 sensors at 1-minute intervals

Sensor No.	S1	S2	S3	S4	S5	S6	S7	S8	S9	S10	S11	S12	S13	S14	S15	S16	S17	S18	S19	S20	S21	S22	S23
RMSE (ppm)	13.0	16.6	17.2	17.9	23.2	13.4	9.3	19.2	21.4	14.3	31.8	31.1	13.3	14.2	19.7	23.8	16.3	17.4	14.6	23.8	16.9	22.3	20.6
R^2	0.9981	0.9982	0.9975	0.9986	0.9979	0.9989	0.9990	0.9983	0.9965	0.9984	0.9969	0.9975	0.9989	0.9984	0.9987	0.9981	0.9974	0.9968	0.9977	0.9973	0.9975	0.9982	0.9975
MBE (ppm)	3.2	1.0	-4.1	13.4	11.3	-5.3	1.4	12.6	-13.5	6.5	-27.0	-22.2	-3.1	5.0	14.7	-19.8	0.5	-5.1	-0.8	6.5	8.2	11.0	-8.9
CV (%)	1.6	2.1	2.1	1.4	2.6	1.6	1.1	1.8	2.3	1.6	2.3	2.9	1.6	1.6	1.6	1.8	2.0	2.2	1.9	2.8	1.9	2.3	2.5
SEE (ppm)	12.4	12.2	14.3	10.7	13.2	9.5	8.9	11.7	17.0	11.5	15.8	14.4	9.3	11.5	10.2	12.5	14.7	16.3	13.7	15.0	14.4	12.3	14.4
<-30 ppm - 3%	0.00%	0.00%	0.25%	0.25%	1.24%	0.00%	0.00%	0.25%	0.25%	0.00%	3.23%	4.55%	0.00%	0.25%	0.00%	0.66%	0.17%	1.24%	0.17%	0.25%	0.00%	0.25%	1.24%
>+30 ppm + 3%	0.25%	0.25%	0.58%	0.00%	0.00%	0.00%	0.17%	0.00%	0.25%	0.17%	0.25%	0.00%	0.25%	0.00%	0.33%	0.17%	1.16%	0.25%	0.83%	3.73%	0.41%	3.73%	0.25%

Table S7. Co-location period 2. Performance metrics pre-normalisation for 23 co-located CO_2 sensors at 1-minute intervals

Sensor No.	S1	S2	S3	S4	S5	S6	S7	S8	S9	S10	S11	S12	S13	S14	S15	S16	S17	S18	S19	S20	S21	S22	S23
RMSE (ppm)	14.6	15.4	23.8	23.3	29.4	22.0	12.7	25.2	24.2	23.9	38.6	26.0	16.0	18.4	28.2	34.2	12.7	25.4	15.0	21.3	20.1	18.3	13.9
R^2	0.9987	0.9987	0.9968	0.9982	0.9988	0.9988	0.9990	0.9989	0.9992	0.9977	0.9987	0.9984	0.9991	0.9989	0.9989	0.9988	0.9992	0.9980	0.9991	0.9983	0.9993	0.9985	0.9991
MBE (ppm)	-0.8	-5.6	-6.3	15.4	25.4	16.6	0.6	20.7	-21.5	14.1	-35.0	-19.9	-10.5	12.0	24.4	-31.3	-5.7	-17.7	-8.9	12.5	15.4	8.9	-6.7
CV (%)	1.1	1.0	1.6	1.5	1.2	1.1	1.0	1.2	1.0	1.4	1.4	1.5	1.1	1.1	1.2	1.2	0.8	1.5	1.0	1.4	1.0	1.2	0.9
SEE (ppm)	14.4	14.5	23.1	17.0	13.9	13.8	12.7	13.7	11.3	19.3	14.4	16.5	12.2	13.2	13.5	14.0	11.5	18.0	12.1	16.5	10.9	15.8	12.3
<-30 ppm - 3%	0.00%	0.00%	0.92%	0.00%	0.00%	0.00%	0.00%	0.00%	0.00%	0.00%	2.45%	0.61%	0.00%	0.00%	0.00%	1.22%	0.00%	0.92%	0.00%	0.00%	0.00%	0.00%	0.00%
>+30 ppm + 3%	0.00%	0.00%	0.61%	2.14%	3.06%	0.92%	0.00%	0.92%	0.00%	2.14%	0.00%	0.00%	0.00%	0.61%	0.92%	0.00%	0.00%	0.00%	0.00%	0.61%	0.00%	0.00%	0.00%

Table S8. Co-location period 3. Performance metrics pre-normalisation for 23 co-located CO_2 sensors at 1-minute intervals

Sensor No.	S1	S2	S3	S4	S5	S6	S7	S8	S9	S10	S11	S12	S13	S14	S15	S16	S17	S18	S19	S20	S21	S22	S23
RMSE (ppm)	14.1	9.9	16.3	15.6	25.2	29.8	9.0	21.0	18.9	11.9	28.2	24.0	16.1	12.5	16.5	22.3	12.5	27.1	12.3	11.2	12.7	10.5	13.2
R^2	0.9987	0.9992	0.9992	0.9995	0.9993	0.9991	0.9993	0.9991	0.9995	0.9992	0.9993	0.9994	0.9993	0.9990	0.9994	0.9991	0.9993	0.9984	0.9991	0.9991	0.9993	0.9992	0.9991
MBE (ppm)	-5.6	2.1	-12.4	14.1	23.3	28.2	-0.5	18.3	-16.4	7.0	-25.4	-21.7	-12.5	3.8	14.1	-18.8	-7.6	22.1	-5.8	3.0	8.6	3.2	-7.7
CV (%)	1.6	1.2	1.3	1.1	1.4	1.5	1.1	1.5	1.1	1.3	1.5	1.2	1.3	1.5	1.3	1.5	1.2	2.1	1.3	1.4	1.3	1.3	1.3
SEE (ppm)	12.5	9.8	9.5	7.5	9.5	10.2	8.9	10.6	8.1	9.8	9.4	8.6	9.2	10.8	8.6	10.5	9.4	13.9	10.4	10.4	9.2	10.0	10.3
<-30 ppm - 3%	0.10%	0.10%	0.25%	0.00%	0.00%	0.00%	0.10%	0.00%	0.00%	0.10%	0.35%	0.05%	0.15%	0.00%	0.00%	0.05%	0.10%	0.00%	0.00%	0.00%	0.00%	0.10%	0.10%
>+30 ppm + 3%	0.00%	0.00%	0.00%	0.00%	0.00%	0.00%	0.00%	0.20%	0.00%	0.00%	0.00%	0.00%	0.00%	0.10%	0.00%	0.10%	0.00%	0.10%	0.00%	0.00%	0.00%	0.00%	0.00%

Table S9. Bias Adjustment Test on Co-location period 1.

Sensor No.	S1	S2	S3	S4	S5	S6	S7	S8	S9	S10	S11	S12	S13	S14	S15	S16	S17	S18	S19	S20	S21	S22	S23
RMSE (ppm)	15.5	16.6	18.9	11.1	22.8	33.2	9.5	14.8	17.8	12.4	17.8	22.4	16.7	13.1	12.4	14.2	18.6	26.6	16.0	22.9	14.4	20.7	18.9
R^2	0.9981	0.9982	0.9975	0.9986	0.9979	0.9989	0.9990	0.9983	0.9965	0.9984	0.9969	0.9975	0.9989	0.9984	0.9987	0.9981	0.9974	0.9968	0.9977	0.9973	0.9975	0.9982	0.9975
MBE (ppm)	7.9	-0.3	7.2	-1.1	-12.5	-32.1	1.5	-6.3	3.3	-1.8	-0.5	-1.0	8.9	-0.2	-1.1	0.4	7.6	-21.9	5.1	1.9	-1.6	6.7	-1.6
CV (%)	1.7	2.1	2.0	1.4	2.6	1.6	1.1	1.8	2.2	1.6	2.3	2.9	1.6	1.6	1.6	1.8	1.9	2.2	1.8	2.7	1.9	2.3	2.5
SEE (ppm)	12.4	12.2	14.3	10.7	13.2	9.5	8.9	11.7	17.0	11.5	15.8	14.4	9.3	11.5	10.2	12.5	14.7	16.3	13.7	15.0	14.4	12.3	14.4

<-30 ppm - 3%	0.00%	0.00%	0.25%	0.25%	1.82%	2.65%	0.00%	0.50%	0.25%	0.00%	0.91%	2.07%	0.00%	0.33%	0.00%	0.00%	0.17%	2.32%	0.17%	0.25%	0.00%	0.25%	1.08%
>+30 ppm + 3%	0.25%	0.25%	1.08%	0.00%	0.00%	0.00%	0.17%	0.00%	0.41%	0.17%	0.25%	0.00%	0.25%	0.00%	0.17%	0.17%	1.16%	0.17%	0.83%	3.56%	0.25%	2.73%	0.25%

Table S10. Bias Adjustment Test on Co-location period 2.

Sensor No.	S1	S2	S3	S4	S5	S6	S7	S8	S9	S10	S11	S12	S13	S14	S15	S16	S17	S18	S19	S20	S21	S22	S23
RMSE (ppm)	14.7	16.0	23.3	17.5	16.0	14.3	12.7	15.0	12.7	20.7	18.7	17.1	12.3	16.0	17.4	18.6	11.5	34.6	13.0	19.1	14.6	16.2	12.4
R^2	0.9987	0.9987	0.9968	0.9982	0.9988	0.9988	0.9990	0.9989	0.9992	0.9977	0.9987	0.9984	0.9991	0.9989	0.9989	0.9988	0.9992	0.9980	0.9991	0.9983	0.9993	0.9985	0.9991
MBE (ppm)	1.3	-7.5	2.8	1.3	6.4	0.7	0.1	4.3	-6.4	7.0	-9.3	1.8	-1.8	7.6	9.8	-12.3	-1.4	-29.8	-5.2	7.9	6.7	2.6	1.2
CV (%)	1.1	1.1	1.8	1.3	1.1	1.1	1.0	1.1	0.9	1.5	1.3	1.3	0.9	1.0	1.1	1.1	0.9	1.5	1.0	1.4	1.0	1.2	1.0
SEE (ppm)	14.4	14.5	23.1	17.0	13.9	13.8	12.7	13.7	11.3	19.3	14.4	16.5	12.2	13.2	13.5	14.0	11.5	18.0	12.1	16.5	10.9	15.8	12.3
<-30 ppm - 3%	0.00%	0.00%	0.92%	0.00%	0.00%	0.00%	0.00%	0.00%	0.00%	0.00%	0.00%	0.00%	0.00%	0.00%	0.00%	0.00%	0.00%	1.53%	0.00%	0.31%	0.00%	0.31%	0.00%
>+30 ppm + 3%	0.00%	0.00%	0.61%	0.00%	0.00%	0.00%	0.00%	0.00%	0.00%	0.92%	0.00%	0.00%	0.00%	0.00%	0.61%	0.00%	0.00%	0.00%	0.00%	0.00%	0.00%	0.00%	0.00%

Table S11. Bias Adjustment Test on Co-location period 3.

Sensor No.	S1	S2	S3	S4	S5	S6	S7	S8	S9	S10	S11	S12	S13	S14	S15	S16	S17	S18	S19	S20	S21	S22	S23
RMSE (ppm)	14.7	9.9	14.9	10.7	19.6	29.8	9.1	15.9	13.4	10.4	17.7	15.9	14.6	12.1	11.2	15.0	12.4	29.7	11.9	11.0	10.5	10.4	11
R^2	0.9987	0.9992	0.9992	0.9995	0.9993	0.9991	0.9993	0.9991	0.9995	0.9992	0.9993	0.9994	0.9993	0.9990	0.9994	0.9991	0.9993	0.9984	0.9991	0.9991	0.9993	0.9992	0.9991
MBE (ppm)	-5.9	3.0	-9.7	8.9	17.9	29.2	-0.3	12.9	-9.1	4.3	-12.2	-11.6	-9.7	1.7	7.7	-8.4	-6.5	26.2	-3.9	0.4	5.1	-0.6	-3.3
CV (%)	1.6	1.2	1.3	1.0	1.4	1.5	1.1	1.5	1.1	1.3	1.5	1.1	1.2	1.5	1.2	1.5	1.2	2.1	1.3	1.4	1.3	1.3	1.3
SEE (ppm)	12.5	9.8	9.5	7.5	9.5	10.2	8.9	10.6	8.1	9.8	9.4	8.6	9.2	10.8	8.6	10.5	9.4	13.9	10.4	10.4	9.2	10.0	10.3
<-30 ppm - 3%	0.05%	0.10%	0.15%	0.00%	0.00%	0.00%	0.10%	0.00%	0.00%	0.10%	0.10%	0.00%	0.10%	0.00%	0.00%	0.00%	0.10%	0.00%	0.00%	0.00%	0.05%	0.10%	0.10%
>+30 ppm + 3%	0.00%	0.00%	0.00%	0.00%	0.00%	0.00%	0.00%	0.15%	0.00%	0.00%	0.00%	0.00%	0.00%	0.10%	0.00%	0.10%	0.00%	0.10%	0.00%	0.00%	0.00%	0.00%	0.00%

Table S12. Regression Adjustment Test on Co-location period 1.

Sensor No.	S1	S2	S3	S4	S5	S6	S7	S8	S9	S10	S11	S12	S13	S14	S15	S16	S17	S18	S19	S20	S21	S22	S23

RMSE (ppm)	15.4	17.0	19.3	11.7	24.8	33.2	9.5	16.1	18.0	11.9	16.1	21.4	17.0	15.5	10.8	16.4	18.7	29.1	16.5	20.7	14.9	19.7	19.1
R^2	0.9981	0.9982	0.9975	0.9986	0.9979	0.9989	0.9990	0.9983	0.9965	0.9984	0.9969	0.9975	0.9989	0.9984	0.9987	0.9981	0.9974	0.9968	0.9977	0.9973	0.9974	0.9981	0.9975
MBE (ppm)	7.5	-0.7	6.9	-1.0	-11.9	-31.9	1.3	-6.0	2.8	-1.7	-1.8	-1.5	8.6	0.4	-0.6	-0.6	7.4	-25.0	4.7	2.3	-1.1	6.8	-1.8
CV (%)	1.6	2.2	2.1	1.4	2.9	1.7	1.1	2.0	2.2	1.5	2.1	2.7	1.7	1.9	1.4	2.1	2.0	2.2	1.9	2.4	1.9	2.1	2.5
SEE (ppm)	12.4	12.2	14.3	10.8	13.2	9.5	8.9	11.8	17.1	11.5	15.8	14.4	9.3	11.5	10.2	12.5	14.7	16.3	13.8	15.0	14.5	12.3	14.4
<-30 ppm - 3%	0.00%	0.00%	0.25%	0.25%	2.73%	2.81%	0.00%	0.66%	0.25%	0.00%	0.33%	1.90%	0.00%	0.33%	0.00%	0.00%	0.17%	2.24%	0.17%	0.25%	0.00%	0.25%	1.08%
>+30 ppm + 3%	0.25%	0.25%	0.99%	0.00%	0.00%	0.00%	0.17%	0.00%	0.41%	0.17%	0.25%	0.00%	0.41%	0.00%	0.17%	0.58%	1.24%	0.17%	0.99%	2.73%	0.25%	2.32%	0.25%

Table S13. Regression Adjustment Test on Co-location period 2.

Sensor No.	S1	S2	S3	S4	S5	S6	S7	S8	S9	S10	S11	S12	S13	S14	S15	S16	S17	S18	S19	S20	S21	S22	S23
RMSE (ppm)	15.0	19.9	23.1	17.3	18.0	14.5	12.7	15.4	12.2	19.9	14.5	22.3	13.4	14.8	14.0	17.7	12.0	25.4	13.3	17.4	12.5	17.4	15.6
R^2	0.9987	0.9987	0.9968	0.9983	0.9988	0.9988	0.9990	0.9989	0.9992	0.9977	0.9987	0.9984	0.9991	0.9989	0.9989	0.9988	0.9992	0.9981	0.9991	0.9983	0.9993	0.9985	0.9991
MBE (ppm)	3.9	-12.9	1.6	1.3	9.6	1.7	0.1	5.5	-4.8	3.6	2.2	14.0	-4.4	6.0	3.0	-10.7	-3.5	-16.9	-5.4	-3.2	4.3	-5.7	8.3
CV (%)	1.1	1.2	1.8	1.3	1.2	1.1	1.0	1.1	0.9	1.5	1.1	1.3	1.0	1.0	1.1	1.1	0.9	1.5	1.0	1.3	0.9	1.3	1.0
SEE (ppm)	14.5	14.5	23.1	17.0	13.9	13.8	12.7	13.6	11.3	19.3	14.4	16.5	12.1	13.2	13.6	14.0	11.5	17.9	12.1	16.6	10.9	15.8	12.4
<-30 ppm - 3%	0.00%	0.00%	0.92%	0.00%	0.00%	0.00%	0.00%	0.00%	0.00%	0.00%	0.00%	0.00%	0.00%	0.00%	0.00%	0.00%	0.00%	0.31%	0.00%	0.31%	0.00%	0.61%	0.00%
>+30 ppm + 3%	0.00%	0.00%	0.61%	0.00%	0.00%	0.00%	0.00%	0.00%	0.00%	0.92%	0.00%	0.61%	0.00%	0.00%	0.00%	0.00%	0.00%	0.00%	0.00%	0.00%	0.00%	0.00%	0.00%

Table S14. Regression Adjustment Test on Co-location period 3.

Sensor No.	S1	S2	S3	S4	S5	S6	S7	S8	S9	S10	S11	S12	S13	S14	S15	S16	S17	S18	S19	S20	S21	S22	S23
RMSE (ppm)	15.9	10.9	13.8	8.2	15.5	29.9	9.4	12.2	8.5	10.1	9.8	13.2	12.9	13.5	9.1	11.4	12.2	30.5	11.4	13.0	9.5	13.0	13.2
R^2	0.9987	0.9992	0.9992	0.9995	0.9993	0.9991	0.9993	0.9991	0.9995	0.9992	0.9993	0.9994	0.9993	0.9990	0.9994	0.9991	0.9993	0.9984	0.9991	0.9991	0.9993	0.9992	0.9991
MBE (ppm)	-8.1	4.5	-5.8	0.4	7.8	29.4	-0.9	3.8	-1.2	0.7	0.8	-3.3	-6.3	-2.8	0.0	4.0	-5.5	28.6	-2.6	-0.3	0.1	-4.1	-1.3
CV (%)	1.6	1.3	1.5	1.0	1.8	1.5	1.1	1.6	1.0	1.3	1.2	1.6	1.3	1.6	1.1	1.4	1.3	1.9	1.3	1.6	1.2	1.5	1.6
SEE (ppm)	12.5	9.8	9.5	7.6	9.5	10.3	8.9	10.6	8.1	9.9	9.4	8.6	9.2	10.8	8.6	10.5	9.4	13.9	10.5	10.4	9.2	10.0	10.3
<-30 ppm - 3%	0.15%	0.10%	0.15%	0.00%	0.00%	0.00%	0.10%	0.00%	0.00%	0.10%	0.00%	0.00%	0.10%	0.00%	0.00%	0.00%	0.10%	0.00%	0.00%	0.00%	0.05%	0.49%	0.00%

>+30 ppm + 3%	0.00%	0.00%	0.00%	0.00%	0.00%	0.00%	0.00%	0.10%	0.00%	0.00%	0.00%	0.00%	0.00%	0.10%	0.00%	0.10%	0.00%	0.10%	0.00%	0.00%	0.00%	0.00%	0.00%

Table S15. Regression coefficients and performance metrics pre- and post-normalisation based on all co-location data

			RMSE (ppm)		R^2		MBE (ppm)		CV (%)		SEE (ppm)		Out of Range Readings	
Sensor ID	Slope (a)	Intercept (b)	Pre	Post	Pre	Post	Pre	Post	Pre	Post	Pre	Post	Pre	Post
S1	0.996	1.38	13.8	13.6	0.9986	0.9986	-2	0	1.59	1.63	13.6	13.6	0.11%	0.08%
S2	1.005	-3.39	13.1	12.8	0.9988	0.9988	1	0	1.53	1.53	12.8	12.8	0.14%	0.14%
S3	1.002	-11.11	17.4	14.8	0.9984	0.9984	-9	0	1.72	1.73	14.7	14.8	0.31%	0.25%
S4	1.001	13.00	17.2	10.2	0.9992	0.9992	14	0	1.27	1.20	10.2	10.2	0.08%	0.08%
S5	0.998	20.71	25.0	15.8	0.9981	0.9981	19	0	1.96	1.94	15.8	15.8	0.42%	0.56%
S6	1.000	16.10	24.7	19.1	0.9973	0.9973	16	0	2.34	2.33	19.1	19.1	0.00%	0.08%
S7	1.000	0.09	9.5	9.5	0.9993	0.9993	0	0	1.11	1.12	9.5	9.5	0.11%	0.11%
S8	1.001	16.06	20.8	12.7	0.9988	0.9988	17	0	1.60	1.54	12.7	12.7	0.20%	0.28%
S9	0.995	-11.73	20.3	12.5	0.9988	0.9988	-16	0	1.57	1.50	12.4	12.5	0.17%	0.17%
S10	1.007	1.77	14.2	11.8	0.9990	0.9990	7	0	1.44	1.42	11.8	11.8	0.11%	0.11%
S11	0.978	-8.34	30.5	12.5	0.9988	0.9988	-27	0	1.82	1.53	12.5	12.5	1.57%	0.20%
S12	0.982	-6.59	26.8	14.5	0.9984	0.9984	-22	0	1.92	1.74	14.5	14.5	1.63%	0.56%
S13	1.003	-11.27	15.2	12.0	0.9989	0.9989	-9	0	1.46	1.41	12.0	12.0	0.14%	0.06%
S14	1.006	0.25	13.7	12.5	0.9988	0.9988	5	0	1.51	1.49	12.5	12.5	0.14%	0.17%
S15	1.014	3.81	19.0	10.0	0.9992	0.9992	15	0	1.41	1.19	10.0	10.0	0.06%	0.06%
S16	0.992	-13.91	24.2	12.9	0.9988	0.9988	-20	0	1.61	1.55	12.8	12.9	0.48%	0.11%
S17	1.003	-6.89	14.0	13.0	0.9987	0.9987	-5	0	1.53	1.51	13.0	13.0	0.22%	0.25%
S18	0.970	34.60	24.1	20.2	0.9969	0.9969	9	0	2.73	2.47	20.2	20.2	0.62%	0.45%
S19	0.998	-2.67	13.4	12.7	0.9988	0.9988	-4	0	1.50	1.49	12.6	12.7	0.11%	0.11%
S20	1.020	-11.47	17.5	14.8	0.9984	0.9984	5	0	1.95	1.72	14.7	14.8	0.59%	0.17%
S21	1.007	2.95	15.0	11.6	0.9990	0.9990	9	0	1.49	1.42	11.6	11.6	0.08%	0.11%
S22	1.013	-4.82	16.2	13.9	0.9986	0.9986	6	0	1.72	1.61	13.8	13.9	0.45%	0.42%
S23	0.990	0.33	16.2	13.7	0.9986	0.9986	-8	0	1.72	1.68	13.7	13.7	0.53%	0.34%
